# BTK Inhibition in Hematology: From CLL/SLL to Emerging Applications Across B-Cell and Immune Disorders

**DOI:** 10.3390/biom16010123

**Published:** 2026-01-12

**Authors:** Andrea Duminuco, Paola De Luca, Gaia Stanzione, Laura Anastasia Caruso, Giulio Lavenia, Salvatore Scarso, Bruno Garibaldi, Fanny Erika Palumbo, Calogero Vetro, Giuseppe Alberto Palumbo

**Affiliations:** 1 Hematology with BMT Unit, A.O.U. Policlinico “G. Rodolico-S. Marco”, 95123 Catania, Italy; andrea.duminuco@unict.it (A.D.); paodeluca37@gmail.com (P.D.L.); gaiastanzione97@gmail.com (G.S.); laura75.caruso@libero.it (L.A.C.); giuliolavenia1999@gmail.com (G.L.); brunga93@gmail.com (B.G.); fannypalumbo@gmail.com (F.E.P.); 2 Department of Infectious, Tropical Diseases and Microbiology, IRCCS Sacro Cuore Don Calabria Hospital, 37024 Negrar di Valpolicella, Italy; salvatorescarso31@gmail.com; 3 Hematology and Bone Marrow Transplantation Unit, Hospital of Bolzano (SABES-Azienda Sanitaria dell’Alto Adige), Teaching Hospital of Paracelsus Medical University, 39100 Bolzano, Italy; gerovetro@gmail.com; 4 Department of Medical Surgical Sciences and Advanced Technologies “G.F. Ingrassia”, University of Catania, 95123 Catania, Italy

**Keywords:** Bruton’s tyrosine kinase, inhibitors, chronic lymphocytic leukemia, covalent and non-covalent BTKi

## Abstract

BTK (Bruton’s tyrosine kinase) has become a key therapeutic target across several hematologic diseases, beginning with its original use in CLL/SLL. As a central mediator of B-cell receptor signaling and microenvironment interactions, BTK supports survival, proliferation, and trafficking in multiple mature B-cell malignancies (mantle cell lymphoma, marginal zone lymphoma, Waldenström macroglobulinemia, and other indolent/aggressive lymphomas) and in selected immune-mediated conditions such as chronic graft-versus-host disease. Covalent BTK inhibitors (ibrutinib, acalabrutinib, and zanubrutinib) irreversibly bind the C481 residue and have produced high response rates and durable disease control, often replacing chemoimmunotherapy in the relapsed setting and, for some entities, even in the first line. Differences in kinase selectivity lead to different safety profiles: second-generation covalent agents generally maintain efficacy while reducing significant off-target toxicities, especially atrial fibrillation and hypertension. Resistance to covalent BTK inhibitors most commonly develops through BTK C481 substitutions and activating PLCG2 mutations, with other kinase-domain variants increasingly recognized. Non-covalent BTK inhibitors (e.g., pirtobrutinib) bind BTK independently of C481, can overcome classic C481-mediated resistance, and extend BTK pathway targeting into later lines of therapy. Overall, BTK inhibition has evolved into a versatile platform enabling long-term, often chemo-free management strategies.

## 1. Introduction

Bruton’s tyrosine kinase (BTK) has emerged first as a pivotal therapeutic target in chronic lymphocytic leukemia/small lymphocytic lymphoma (CLL/SLL), reshaping management from conventional chemoimmunotherapy toward chronic, pathway-directed inhibition. BTK is a central node in B-cell receptor signaling and microenvironmental crosstalk, and its inhibition disrupts survival and trafficking cues across a broad spectrum of hematological conditions [1]. In CLL, covalent BTK inhibitors have consistently produced deep and durable disease control, including in high-risk cohorts, and have therefore been widely adopted as standards of care in both first-line and relapsed/refractory disease [2,3]. Current international guidelines, including NCCN, now prominently position BTKi-based regimens across lines of therapy: BTKi, alone or in combination with venetoclax and/or obinutuzumab, are listed as preferred first-line options alongside venetoclax–obinutuzumab, with treatment selection increasingly individualized according to disease biology, comorbidity profile (with special attention to cardiovascular risk and bleeding diathesis), patient preferences for continuous versus fixed-duration strategies, and health-system factors such as access to minimal residual disease (MRD) testing and infusion resources [4,5,6,7]. As randomized datasets mature and MRD-driven concepts are tested in BTKi monotherapy and combination trials, the positioning of BTKi relative to venetoclax-based, time-limited regimens and anti-CD20-containing combinations is being progressively refined, further differentiating continuous single-agent BTK inhibition from time-limited, MRD-oriented approaches in both frontline and relapsed/refractory settings.

Within this therapeutic framework, CLL/SLL remains the most common leukemia of adults in Western countries, with a clear predominance of elderly, multimorbid patients in routine practice. Treatment indications are still defined by the iwCLL criteria, which mandate a “watch-and-wait” strategy for asymptomatic early-stage disease and recommend initiation of therapy only when specific clinical triggers emerge, such as progressive marrow failure with worsening cytopenias, symptomatic or bulky lymphadenopathy or splenomegaly, constitutional “B” symptoms, or rapid lymphocyte kinetics suggestive of accelerated disease biology [8]. In contemporary practice, molecular risk assessment is systematically integrated into baseline work-up. TP53 disruption (either 17p deletion or TP53 mutation) and IGHV mutational status are key determinants for both prognostication and treatment selection, because patients with TP53 aberrations or unmutated IGHV derive limited benefit and inferior long-term outcomes from traditional chemoimmunotherapy [9].

Most patients present with asymptomatic lymphocytosis identified incidentally, and only 20–30% require treatment at diagnosis; however, the clinical course is highly heterogeneous, ranging from indolent disease that may never require therapy to rapidly progressive forms with symptomatic lymphadenopathy, cytopenias, infections, or transformation into more aggressive diseases. The impact on quality of life (QoL) reflects the chronic, often lifelong course of this disease in an older, comorbid population (approximately 95% of patients have at least one comorbid condition), complicating disease management and daily functioning. Disease progression can lead to constitutional “B” symptoms, symptomatic lymphadenopathy or splenomegaly, cytopenias (anemia, thrombocytopenia), recurrent infections related to disease- and treatment-induced immunosuppression, and autoimmune complications such as autoimmune hemolytic anemia or immune thrombocytopenia, all of which further impair QoL and frequently require hospitalization [10,11,12,13,14,15]. The traditional “watch-and-wait” approach in indolent B-cell hematological conditions means that many patients live for years with a known but untreated malignancy, undergoing regular monitoring and facing persistent uncertainty about when treatment will become necessary, which adds psychological burden for patients and caregivers. Advanced age and comorbidities increase susceptibility to treatment-related toxicities, frailty, and functional decline, making individualized therapeutic strategies essential to balance disease control with maintenance of autonomy and daily functioning. In this context, the choice among continuous BTKi, time-limited venetoclax-based regimens, or other targeted approaches must be guided not only by disease biology but also by comorbidity burden, frailty, and patient preferences. Finally, drug–drug interactions with CYP3A inhibitors or inducers, proton pump inhibitors, and anticoagulants are common in this population. They are particularly relevant for BTKi, necessitating careful evaluation to avoid dose reductions, interruptions, or premature discontinuations [16].

## 2. BTKi: Mechanism of Action and Resistance Biology

BTK sits at a critical bottleneck of B-cell receptor (BCR) signaling, and a mechanistic understanding of BTK biology is essential to explain why BTK inhibition has been so transformative [17]. In resting B cells, surface immunoglobulin complexes (mIg–CD79A/B) couple antigen recognition to intracellular signaling via ITAM phosphorylation by SRC-family kinases (primarily LYN), which recruits and activates SYK [18]. Activated SYK then propagates the signal through multiple scaffolds, including BLNK/SLP-65, bringing BTK and PLCγ2 into proximity at the inner leaflet of the plasma membrane. BTK itself is activated through a two-step process. PI3K-generated PIP3 engages the BTK pleckstrin homology domain, anchoring the kinase to the membrane. In contrast, transphosphorylation of the activation loop tyrosine (Y551) by SRC/SYK family kinases, followed by BTK autophosphorylation at Y223, stabilizes the active conformation. In its active state, BTK phosphorylates PLCγ2, which hydrolyzes PIP2 to generate IP3 and DAG, triggering calcium flux, PKCβ activation, and the assembly of downstream signaling hubs controlling NF-κB, NFAT, and MAPK/ERK pathways [19]. Parallel outputs include cytoskeletal remodeling, integrin activation, and transcriptional programs that support survival, proliferation, and migration. In CLL/SLL and other mature B-cell neoplasms, this axis is chronically engaged not only by autonomous or tonic BCR signaling but also by microenvironmental inputs, including BAFF, chemokines (e.g., CXCL12, CXCL13), integrin ligands, and stromal or nurse-like cell interactions, effectively wiring BTK into both antigen-dependent and antigen-independent survival circuits [20,21,22,23].

**Figure 1:**
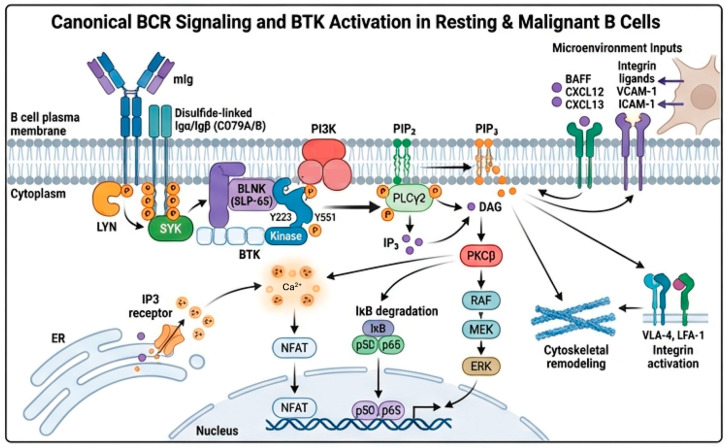
The BCR signaling pathway, as described in the text.

The BCR signaling pathway is illustrated in Figure 1.

Covalent BTK inhibitors exploit this nodal position by irreversibly targeting the cysteine 481 (C481) residue within the ATP-binding pocket. By forming a covalent bond at C481, ibrutinib, acalabrutinib, and zanubrutinib “lock” BTK in an inhibited state long after free drug has cleared from plasma, leading to sustained suppression of proximal BCR signaling. Functionally, this translates into blunted PLCγ2 activation, reduced IP3-dependent calcium mobilization, impaired PKCβ and IKK activation, and attenuation of canonical NF-κB and ERK signaling, thereby curtailing transcriptional programs that maintain B-cell survival and proliferation [23]. At the cellular level, BTK inhibition also interferes with chemokine-mediated trafficking (e.g., CXCR4/CXCL12 and CXCR5/CXCL13 axes), integrin-mediated adhesion (particularly VLA-4/VCAM-1), and retention of leukemic cells within lymphoid niches. This disruption of microenvironmental crosstalk underlies the characteristic pharmacodynamic phenotype of covalent BTKi: rapid egress of tumor cells from nodes and marrow into the peripheral blood (“redistribution lymphocytosis”), followed by progressive nodal shrinkage and, over time, contraction of the circulating clone as survival signals are withdrawn [24,25].

Despite sharing this core mechanism, covalent BTKi differ in kinase selectivity and off-target engagement. Ibrutinib, the first-in-class agent, inhibits not only BTK but also multiple TEC-family kinases (TEC, ITK), EGFR, and kinases implicated in platelet function and cardiac electrophysiology [26,27]. These off-target interactions likely contribute to its distinctive toxicity profile, including atrial fibrillation, hypertension, bleeding, and perturbation of T-cell and NK-cell function. Second-generation covalent inhibitors, acalabrutinib and zanubrutinib, were engineered with greater selectivity for BTK and reduced activity against TEC, EGFR, and other off-target kinases, enabling complete and more sustained BTK occupancy in lymphoid tissues while generally lowering the incidence of cardiovascular and hemostatic adverse events observed in head-to-head trials [28]. From a biological standpoint, these agents more cleanly dissect BTK-dependent BCR and microenvironmental signaling from off-target kinase networks, providing a clearer view of “pure” BTK pathway inhibition in human disease.

Resistance to covalent BTKi is, in large part, a story of how malignant B cells rewire this signaling architecture under therapeutic pressure. The prototypical mechanism involves substitution of C481, most commonly C481S, which abrogates covalent inhibitors’ ability to form an irreversible bond. Structurally, the cysteine-to-serine change preserves overall kinase function but converts the covalent interaction into a weaker, reversible one, allowing ATP to outcompete the drug at clinically achievable concentrations [21,29]. This single amino-acid change is sufficient to restore BTK catalytic activity and re-enable BCR signaling despite continued drug exposure. In parallel, gain-of-function mutations in PLCG2 can render PLCγ2 partially or fully autonomous, allowing downstream calcium flux and NF-κB activation to proceed even when BTK itself is inhibited [21,22,30,31]. Together, BTK C481 substitutions and activating PLCG2 variants define a canonical “BTK-proximal” resistance paradigm, in which signaling is restored either at the level of the kinase or immediately downstream.

Larger sequencing cohorts and time-resolved genomic analyses have revealed that this resistance landscape is more heterogeneous than initially appreciated. Distinct mutational spectra emerge under ibrutinib versus acalabrutinib exposure, with different frequencies of C481 substitutions and PLCG2 lesions, suggesting subtle differences in clonal selection under each inhibitor [32]. Additionally, non-C481 BTK-domain mutations have been identified at residues within the gatekeeper region (e.g., T474) and the catalytic/activation loop (e.g., L528W), some of which alter ATP binding, conformational dynamics, or inhibitor affinity without directly disrupting covalent bond formation [33]. These variants can reduce the effective binding of specific BTKi, generating mutation-specific patterns of cross-resistance and sensitivity that are only beginning to be mapped at a structural and functional level.

Non-covalent (reversible) BTKi, such as pirtobrutinib, were rationally designed to circumvent C481 dependence by engaging BTK through a network of non-covalent interactions within the ATP-binding cleft. Because their binding does not rely on the thiol group of C481, these agents retain high potency against BTK harboring C481S and other covalent-resistance substitutions [29]. Their pharmacokinetic and pharmacodynamic properties (such as high affinity, slow off-rates, and relative kinase selectivity) permit sustained BTK pathway suppression even in heavily pretreated patients, including those intolerant or resistant to covalent BTKi [2]. Nonetheless, resistance to non-covalent inhibitors can still emerge through alternative kinase-domain mutations that directly perturb the non-covalent binding interface (for example, at gatekeeper or activation loop residues) or through broader pathway rewiring, such as activation of downstream nodes or parallel survival circuits that bypass BTK entirely [22]. Additional mechanisms, including shifts in lineage programming or engagement of alternative microenvironmental niches, have been proposed in a subset of cases, underscoring that BTK-directed pressure drives a dynamic clonal evolution rather than a single, fixed resistance route [34].

## 3. Evidence-Based in CLL/SLL

### 3.1. First Line

The phase 3 RESONATE-2 trial firstly definitively established single-agent ibrutinib as the first frontline standard for covalent BTK inhibition in older patients. In this study, 269 treatment-naïve patients aged ≥ 65 years without del(17p) were randomized to receive continuous ibrutinib (420 mg once daily) or chlorambucil for up to 12 cycles [35]. After extended follow-up of up to 10 years, the final analysis reported a median progression-free survival (PFS) of 8.9 years with ibrutinib versus 1.3 years with chlorambucil, representing a profound and durable risk reduction for progression or death in favor of ibrutinib. Median overall survival (OS) remained unreached in the ibrutinib arm. In contrast, it was substantially shorter with chlorambucil, and the survival advantage was preserved across genomic risk categories, including patients with unmutated IGHV or other high-risk features [36]. Notably, a clinically meaningful proportion of patients were still receiving ibrutinib at 8–10 years, and long-term tolerability remained acceptable, with no unexpected safety signals emerging over time [36,37]. Collectively, RESONATE-2 not only displaced chlorambucil-based regimens in older or comorbid patients but also provided the quantitative backbone for a chemo-free frontline standard with continuous covalent BTK inhibition.

Two seminal randomized studies then broadened the evidence base across complementary age and fitness strata. In the Alliance A041202 trial, more than 500 previously untreated patients aged ≥ 65 years were randomized to bendamustine–rituximab (BR), ibrutinib monotherapy, or ibrutinib–rituximab. Both ibrutinib-containing regimens significantly prolonged PFS compared with BR, but adding rituximab to ibrutinib did not confer additional PFS benefit, leading to the conclusion that continuous ibrutinib is sufficient in this setting [38]. Long-term analyses showed that the benefit of ibrutinib-based therapy was primarily confined to patients with unmutated IGHV. In contrast, those with mutated IGHV derived less incremental benefit from ibrutinib-based therapy compared with BR [39].

In younger, fit patients (≤70 years), the E1912 trial randomized 529 participants to receive either ibrutinib–rituximab or fludarabine–cyclophosphamide–rituximab (FCR). At approximately three years of follow-up in the primary analysis, 3-year PFS was 89.4% with ibrutinib–rituximab versus 72.9% with FCR (hazard ratio [HR] for progression or death ≈ 0.35), and 3-year OS was 98.8% versus 91.5%, respectively (HR for death ≈ 0.17) [40]. These benefits were observed irrespective of IGHV mutational status and were maintained with longer follow-up. Continuous ibrutinib remained tolerable beyond 5 years in the majority of patients [41]. Together, A041202 and E1912 anchored ibrutinib-based therapy as the preferred standard across both older and younger frontline populations.

The first-line landscape has subsequently diversified with the introduction of second-generation covalent BTK inhibitors. ELEVATE-TN enrolled 535 treatment-naïve patients (median age 70 years, 63% unmutated IGHV, 13.6% with del(17p) and/or TP53 mutation, and 17% with complex karyotype) and randomized them to acalabrutinib–obinutuzumab, acalabrutinib monotherapy, or chlorambucil–obinutuzumab [42]. At six years of follow-up, estimated PFS rates were 78.0% with acalabrutinib–obinutuzumab and 61.5% with acalabrutinib alone, compared with 17.2% with chlorambucil–obinutuzumab; median PFS was not reached in either acalabrutinib-containing arm [43]. The survival and PFS advantages were preserved in high-risk subsets, including patients with del(17p)/TP53 mutation, unmutated IGHV, and complex karyotype, and the long-term safety profile remained favorable, supporting acalabrutinib-based therapy (particularly acalabrutinib–obinutuzumab) as a preferred first-line option for many patients.

SEQUOIA further extended second-generation BTK inhibition into the frontline setting. A cohort of 1479 previously untreated patients without del(17p) was randomized 1:1 to zanubrutinib or BR (241 vs. 238 patients). In the primary analysis, after approximately 2 years of follow-up, 2-year PFS was 85.5% with zanubrutinib compared with 69.5% with BR, confirming a significant PFS benefit with continuous zanubrutinib and an acceptable safety profile [44]. Longer-term data with about 5 years of follow-up have shown sustained PFS separation and 60-month OS rates of about 86% in both arms, with median OS not reached, while PFS remained superior to BR, and atrial fibrillation rates remained low at about 7% [45].

In a nonrandomized cohort dedicated to patients with del(17p), frontline zanubrutinib monotherapy produced robust and durable disease control, with PFS outcomes broadly comparable to those observed with other BTK inhibitors in TP53-aberrant disease, thereby reinforcing the class effect of BTKi in this high-risk population [46].

Looking beyond continuous monotherapy with a single BTKi, a fixed-duration approach has emerged as a viable alternative in first line.

Within this framework, the CAPTIVATE Fixed-Duration (FD) cohort provides a key proof-of-concept for time-limited, all-oral doublet therapy in previously untreated CLL. In this study, patients received three cycles of ibrutinib lead-in followed by twelve cycles of ibrutinib plus venetoclax. The complete response (CR) rate was approximately 55–56%, and undetectable minimal residual disease (uMRD) in peripheral blood was achieved in about 77% of participants. PFS at 24 months approached 95%, and overall survival at the same landmark was roughly 98%. Importantly, these results were maintained even in genomically high-risk subgroups, including those with del(17p), TP53 mutation, or unmutated IGHV, with 36-month PFS estimates of 88–92% and OS exceeding 95% [47,48]. These data support the idea that a finite 15-cycle course of BTKi–venetoclax can deliver deep, durable remissions in both standard- and high-risk disease [9]. Recently, in the CLL17 phase 3 trial for previously untreated CLL (*n* = 909), fixed-duration venetoclax–obinutuzumab or venetoclax–ibrutinib achieved noninferior 3-year PFS compared with continuous ibrutinib (81.1% and 79.4% vs. 81.0%, respectively; both comparisons met noninferiority). After treatment, undetectable MRD in peripheral blood was far higher with fixed-duration therapy (73.3% with venetoclax–obinutuzumab; 47.2% with venetoclax–ibrutinib) than with ibrutinib (0%), while 3-year OS remained high across arms (91.5–96.0%) [49].

AMPLIFY has now extended the fixed-duration paradigm to second-generation BTK inhibitor–based combinations in fit, previously untreated patients. In this phase 3 trial, 867 patients without del(17p) or TP53 mutation were randomized 1:1:1 to acalabrutinib–venetoclax, acalabrutinib–venetoclax–obinutuzumab, or CIT (FCR or BR). At a median follow-up of ~41 months, estimated 36-month PFS was 76.5% with acalabrutinib–venetoclax, 83.1% with the triplet, and 66.5% with CIT; median PFS was not reached in either experimental arm but was 47.6 months in the CIT group (HR for progression or death 0.65 for acalabrutinib–venetoclax vs. CIT and 0.42 for the triplet vs. CIT). Three-year OS remained high across all arms (>85%), and safety was consistent with the known profiles of the individual agents, with neutropenia and infections constituting the main grade ≥ 3 events [50].

MRD-guided BTKi plus venetoclax has further refined the first-line approach. Because AMPLIFY excluded TP53-aberrant patients, an MRD-guided phase 2 trial (NCT03580928) evaluated AVO in high-risk, treatment-naïve CLL enriched for TP53 aberrations: among 72 patients (45 TP53-aberrant), CR with bone marrow uMRD at cycle 16 was 42% (also 42% overall), bone marrow uMRD rates were 71% (TP53-aberrant) and 78% (overall), and with 55.2 months median follow-up, 4-year PFS/OS was 70%/96% in TP53-aberrant patients and 88%/100% in those without TP53 aberration, with infrequent cardiovascular and bleeding events [51]. Secondarily, in the UK FLAIR platform, 786 previously untreated patients were randomized among standard CIT, continuous ibrutinib, or MRD-directed ibrutinib–venetoclax. At five years, approximately 94% of patients treated with ibrutinib–venetoclax were alive without disease progression, compared with ~79% on ibrutinib alone and ~58% on CIT. Moreover, two-thirds of patients in the combination arm achieved undetectable MRD in bone marrow after two years, far exceeding the rates with either comparator [52].

Data from first-line clinical trials are summarized in Table 1.

### 3.2. Subsequent Lines

Cytogenetic and molecular profiling (in particular assessment of del(17p) by FISH, TP53 mutation status by NGS, and CpG-stimulated karyotype) remains mandatory before first-line therapy. It should be repeated at progression when a new line of treatment is considered. IGHV mutational status, once documented, does not need to be repeated. TP53 disruption (del(17p)/TP53 mutation) is present in roughly 10–15% of patients at diagnosis but may increase to 20–30% in the relapsed/refractory (R/R) setting. At progression after BTKi, testing for BTK and PLCG2 mutations can identify specific resistance mechanisms and aid interpretation of patterns of clinical failure [9,53]. However, current guidance emphasizes that BTK/PLCG2 status alone should not trigger a treatment change in the absence of clinical or hematologic progression.

Regimen-specific considerations include whether therapy is continuous or fixed-duration, and whether it is purely oral or combined with intravenous anti-CD20. Continuous BTKi exposes patients to years of treatment, with median PFS in the R/R setting ranging from roughly 38 to 44 months with covalent BTKi in pivotal trials, whereas fixed-duration venetoclax-based combinations, typically administered for 12–24 months, can deliver deep remissions and prolonged treatment-free intervals [9].

Prior treatment history and reasons for discontinuation are equally critical. Progression on a BTKi versus intolerance to BTKi, the depth of response to venetoclax-based therapy, and the duration of the treatment-free interval all influence the likelihood of benefit from re-treatment with the same class, switching to the alternative class (BTKi to BCL2 inhibitor, or vice versa), or moving to a non-covalent BTKi or cellular therapy.

In the R/R setting, the expanding portfolio of BTKi- and BCL2 inhibitor–based regimens allows genuine tailoring of efficacy and safety, and the numerical signals from key trials provide useful benchmarks. ELEVATE-RR randomized high-risk patients, all with del(11q) and/or del(17p), to continuous acalabrutinib 100 mg twice daily versus ibrutinib 420 mg once daily. At a median follow-up of 40.9 months, median PFS was identical in both arms at 38.4 months, formally meeting the noninferiority criterion for acalabrutinib, with a hazard ratio for PFS of approximately 1.00. Importantly, acalabrutinib showed a markedly better cardiovascular profile. Atrial fibrillation or flutter occurred in 9.4% versus 16.0% of patients on ibrutinib, hypertension in 9.4% versus 23.2%, and any-grade bleeding events were also less frequent, with fewer discontinuations due to adverse events, supporting acalabrutinib as a “non-inferior efficacy, superior tolerability” alternative whenever a covalent BTKi is chosen after relapse [54].

ASCEND compared continuous acalabrutinib 100 mg twice daily to the investigator’s choice of idelalisib–rituximab or bendamustine–rituximab in R/R CLL. With a median follow-up of 46.5 months, acalabrutinib significantly improved PFS (median PFS not reached with acalabrutinib versus 16.8 months with comparator, HR = 0.31), with a 42-month PFS rate of 62% with acalabrutinib versus 19% with idelalisib-R/BR [55,56]. This more than 40-percentage-point separation at 3.5 years firmly positions BTKi as the preferred option over PI3K- or chemoimmunotherapy-based regimens for most relapsed patients.

The RESONATE trial, comparing ibrutinib with ofatumumab, provided the initial proof-of-concept for BTKi in R/R disease. With up to 71 months of follow-up, ibrutinib achieved a median PFS of 44.1 months compared with 8.1 months with ofatumumab, conferring an OS benefit and efficacy retained across high-risk subgroups, quantifying the magnitude of the benefit of first-generation BTKi over antibody monotherapy [57,58].

ALPINE directly compared zanubrutinib 160 mg twice daily with ibrutinib 420 mg once daily in R/R CLL. At 42.5 months of follow-up, zanubrutinib demonstrated superior PFS with an HR of 0.68, and 3-year PFS rates of 64.9% versus 54.8% for ibrutinib, alongside a higher overall response rate (85.6% vs. 75.4%). Atrial fibrillation or flutter occurred in 7.1% of zanubrutinib-treated patients, compared with 17.0% with ibrutinib, and overall cardiac events were lower [59,60]. ALPINE thus provided the first head-to-head efficacy advantage over ibrutinib while simultaneously improving cardiac safety.

Pirtobrutinib, evaluated in the phase 3 BRUIN CLL-321 trial, has reshaped options after covalent BTKi. In this study of patients previously treated with covalent BTKi, pirtobrutinib improved PFS and time-to-next-treatment compared with idelalisib–rituximab or bendamustine–rituximab, in a population characterized by multiple prior lines of therapy, substantial comorbidities, and frequent C481-mediated resistance [61]. This randomized evidence complements its 2023 FDA-accelerated approval for CLL/SLL after both a BTKi and a BCL2 inhibitor, operationalizing a modern sequencing framework in which a non-covalent BTKi can serve as an active “bridge” after failure of both covalent BTKi and venetoclax-based therapy.

Data from further-line clinical trials are summarized in Table 2.

## 4. Scenarios in CLL/SLL: From Current Practice to Future Directions

In contemporary clinical practice, BTKi serve as a central therapeutic backbone when durable disease control is required and when patient- and disease-specific features favor continuous oral therapy [9]. At a conceptual level, treatment sequencing can be viewed along lines of therapy, with two dominant frontline philosophies as time-limited BCL-2–based combinations versus continuous covalent BTK inhibition. In the first line, the primary time-limited strategy is venetoclax plus an anti-CD20 monoclonal antibody (obinutuzumab), typically delivered over 12–24 months to achieve deep remission followed by a prolonged treatment-free interval. In fact, CLL14 showed that in 432 elderly/comorbid patients, 12 months of venetoclax–obinutuzumab was clearly superior to chlorambucil–obinutuzumab, with 3-year PFS 81.9% vs. 49.5% (HR ~0.31). About half of patients maintained undetectable MRD 18 months after stopping treatment, establishing this as a one-year, chemo-free standard for this population [62,63,64,65]. The second, fixed-duration approach is venetoclax plus ibrutinib. In clinical practice, it is increasingly considered for younger or fit patients who can tolerate an initial period of intensified monitoring in exchange for a finite course of therapy and a prolonged treatment-free interval.

The alternative is continuous covalent BTKi, with or without an anti-CD20 antibody. Within this paradigm, intolerance is often managed by dose modification or switching to another BTKi rather than abandoning the class.

BTKi are particularly prioritized in patients with TP53 disruption (del(17p) and/or TP53 mutation), in whom responses to chemoimmunotherapy and other approaches are consistently inferior and often short-lived. Within the covalent BTKi class, second-generation agents (acalabrutinib or zanubrutinib) are typically favored because they preserve efficacy while reducing key cardiovascular toxicities compared with ibrutinib, with atrial fibrillation generally in the range of ~7–10% and hypertension around ~10% versus ≥15–20% with ibrutinib in comparative datasets [66,67]. In very elderly or frail individuals, the logistical simplicity of BTKi monotherapy (no infusions, no venetoclax ramp-up, and monitoring primarily focused on blood counts and blood pressure) often makes it an attractive option when tolerability is acceptable. Conversely, when finite therapy and treatment-free intervals are strongly prioritized, and infrastructure permits close early monitoring, time-limited venetoclax-based regimens may be preferred to reduce cumulative exposure to BTKi-associated toxicities and support planned discontinuation.

Among younger, fit patients, BTKi–venetoclax combinations are increasingly incorporated when MRD assessment is feasible, reflecting an evolving preference for depth of response and time-limited strategies that may reduce lifetime exposure to continuous therapy, balanced against the practical requirements of TLS prophylaxis and MRD-driven follow-up. In the relapsed/refractory setting, sequencing is driven primarily by prior class exposure and the reason for discontinuation (progression versus intolerance). Patients who received frontline venetoclax-based fixed-duration therapy commonly transition to a second-generation BTKi at relapse. In contrast, those who began with a BTKi are often treated with venetoclax plus anti-CD20 antibody, a fixed-duration venetoclax-based regimen, or a non-covalent BTKi, such as pirtobrutinib. After failure of a covalent BTKi (particularly when resistance is suspected to be BTK/PLCG2-driven), fixed-duration venetoclax-based therapy becomes a frequent next step.

MURANO evaluated fixed-duration venetoclax 400 mg once daily for two years plus rituximab for six monthly cycles versus bendamustine–rituximab in R/R CLL. At a median follow-up of seven years, venetoclax–rituximab achieved a median PFS of 54.7 months versus 17.0 months with BR and a 7-year PFS of 23.0%. Seven-year overall survival was 69.6% versus 51.0%, respectively. Patients achieving undetectable minimal residual disease had remarkably durable remissions [68,69].

For patients who relapse after both a covalent BTKi and a BCL2 inhibitor, non-covalent BTKi, such as pirtobrutinib, represent a mechanistically rational option supported by randomized evidence. In the third line and beyond, sequencing becomes progressively more complex, with recycling of cBTKi or non-covalent BTKi where appropriate, re-treatment with BCL-2 inhibitor plus anti-CD20 in selected patients with durable prior benefit, and consideration of cellular therapies such as CAR T-cell therapy in fit candidates once both BTK- and BCL2-directed strategies have been exhausted. Across all scenarios, repeat molecular reassessment at progression (del(17p)/TP53 and complex karyotype, with BTK/PLCG2 testing at BTKi failure) integrated with detailed comorbidity profiling supports a pragmatic, individualized BTKi-based strategy.

Secondarily, across all stages, participation in clinical trials is explicitly encouraged for any patient requiring treatment, and it is recognized that widespread use of BTKi plus BCL-2 inhibitor combinations in the frontline setting will further reshape downstream sequencing [70].

For those double-exposed patients (R/R CLL/SLL after both BTKi and BCL2 inhibition), lisocabtagene maraleucel (liso-cel), an anti-CD19 CAR T-cell product, has emerged as a promising salvage option. The phase 2 TRANSCEND CLL 004 trial evaluated liso-cel in this very high-risk population. At dose level 2 (DL2; 100 × 10^6^ CAR-positive T-cells), the complete response/complete response with incomplete count recovery (CR/CRi) rate was 18% (95% CI 9–32), with an overall response rate (ORR) of 43%. Rates of uMRD were striking (63% in peripheral blood and 59% in bone marrow among evaluable patients). The median duration of response was 35.3 months, the median PFS was 11.9 months, and the median OS 30.3 months. Safety was manageable. Grade 3 cytokine release syndrome occurred in 9% of patients and grade 3 neurologic events in 18%, with no treatment-related fatal events reported. In the particularly adverse del(17p)/TP53-mutated subgroup, CR/CRi reached 23% and ORR 47%, indicating that liso-cel can induce meaningful disease control even in the most resistant clones [71].

TRANSCEND CLL 004 also included a separate cohort combining liso-cel with ibrutinib, with early data suggesting potential synergy between ongoing BTK pathway inhibition and CAR T-cell activity. However, the published literature to date focuses primarily on liso-cel monotherapy. A more granular analysis of PFS by best overall response at DL2 showed that, among all responders, the median duration of response was 13.3 months (95% CI 6.0–23.3), with maintained response rates of 51.4% at 12 months and 38.8% at 18 months. In patients achieving complete responses, the median duration of response extended to 17.5 months (95% CI 7.5–not reached), with 12- and 18-month maintained response rates of 57.8% and 48.0%, respectively. In contrast, those with partial responses had a much shorter median duration of response of 2.2 months [72,73].

**Figure 2:**
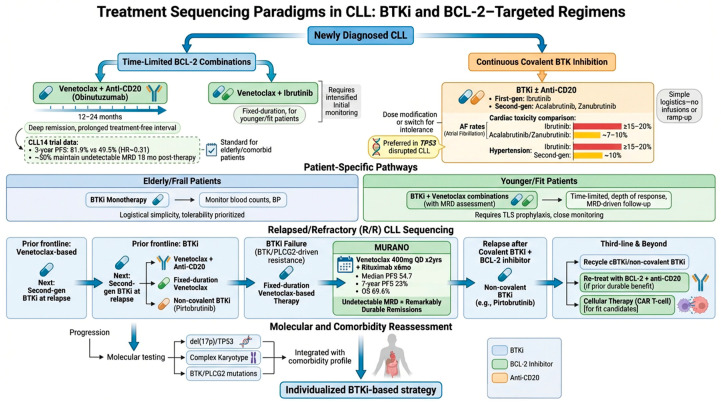
A schematic representation of the therapeutic sequencing paradigms of CLL/SLL.

A graphical representation of SLL/CLL therapeutic scenario is summarized in Figure 2.

## 5. Other Fields of Application for BTKi

Beyond CLL/SLL, BTK inhibition has become an established therapeutic strategy in several B-cell malignancies and immune-mediated disorders, with the most mature data in mantle cell lymphoma (MCL) and marginal zone lymphoma (MZL).

Biologically, MCL is highly dependent on chronic BCR signaling and tonic NF-κB activation, with BTK acting as a key relay between SYK/LYN and downstream NF-κB, PI3K–AKT, and MAPK cascades. This dependence is particularly evident in the classical, nodal, and leukemic non-blastoid variants, in which BTK inhibition rapidly disrupts proliferative and survival signals, inducing characteristic nodal regression and lymphocytosis [74]. In relapsed/refractory MCL, the first single-arm phase II trial of ibrutinib in 111 patients showed an ORR of 68% and a complete response (CR) rate of 21%, with a median PFS of 13.9 months, leading to the initial approval of covalent BTK blockade in this disease [75]. The randomized RAY study subsequently demonstrated superiority of ibrutinib over temsirolimus, with median PFS of 14.6 versus 6.2 months (HR ≈ 0.43), confirming BTKi as the preferred standard in relapsed MCL [76]. More recently, BTK inhibition has moved into the frontline arena. The phase 3 SHINE trial evaluated the addition of ibrutinib to BR induction, followed by rituximab maintenance, in older, previously untreated patients. Ibrutinib–BR significantly prolonged PFS compared with placebo–BR (median PFS ~80.6 vs. 52.9 months; HR ≈ 0.75), with benefit observed across most clinical and biological subgroups, including high-risk patients. However, this came at the cost of increased toxicity (notably infections, atrial fibrillation, and treatment-related deaths), and no overall survival (OS) advantage emerged at the time of reporting, tempering enthusiasm for routine incorporation of ibrutinib into BR backbones in unselected patients [77].

The phase 3 TRIANGLE trial has reshaped frontline management in younger, transplant-eligible patients. Adding ibrutinib to standard R-CHOP/R-DHAP (or R-DHAOx) with post-ASCT maintenance improved 3-year failure-free survival to 88% versus 72% with ASCT-based chemoimmunotherapy alone (HR 0.52). Notably, an ibrutinib-containing regimen without ASCT achieved a 3-year PFS of 86%, and the superiority of ASCT over omission of ASCT in the BTKi era was not demonstrated [78].

Second-generation covalent BTKi further improved this landscape. Acalabrutinib has been studied both as a monotherapy and in combination with chemoimmunotherapy. The ACE-LY-004 phase II trial in R/R MCL reported an ORR above 80%, CR rates around 40%, and a median PFS exceeding 20 months, with a favorable tolerability profile and relatively low rates of atrial fibrillation and major bleeding, supporting its approval in this setting. In the frontline setting, an acalabrutinib-containing regimen plus BR (ECHO/ENRICH program) has shown improved PFS versus BR alone with no OS advantage to date, but with a more manageable cardiovascular profile than ibrutinib-based chemoimmunotherapy, suggesting that second-generation BTK inhibitors may offer a better risk–benefit balance when used in combination induction. Zanubrutinib in study BGB-3111-206 achieved ORR around 84–85% and high CR rates (≈60–70%) with encouraging PFS and a low incidence of atrial fibrillation, supporting these agents as efficacious and better-tolerated options for R/R MCL [79,80,81,82].

More recently, pirtobrutinib has shown clinically meaningful activity in patients who have already failed a covalent BTKi, with response rates in the 50–60% range even in BTK C481-mutated MCL, and is increasingly considered in multiply relapsed, BTKi-exposed disease [83,84,85]. In older, previously untreated mantle cell lymphoma, adding acalabrutinib to BR followed by rituximab maintenance significantly prolonged PFS versus chemoimmunotherapy alone (median 66.4 vs. 49.6 months; HR 0.73), with higher complete response rates (66.6% vs. 53.5%) and benefit preserved across high-risk subgroups. Overall survival was not significantly different at this follow-up, and rates of grade ≥ 3 adverse events were similar between arms (~89%), indicating that the PFS gain was achieved without an apparent increase in severe toxicity [86,87].

In MZL, the single-arm PCYC-1121 phase II trial of ibrutinib enrolled 63 patients with R/R MZL, achieving an ORR of 58% with CR in 10% and a median PFS of 15.7 months, supporting accelerated approval in this indication [88]. The MAGNOLIA study subsequently evaluated zanubrutinib in 68 patients with R/R MZL and reported an ORR of 68% with CR in 26% and an estimated 2-year PFS of ~71%, again with a favorable cardiac safety profile compared with historical ibrutinib data [89].

In Waldenström macroglobulinemia (WM), BTK inhibition is even more tightly anchored to disease biology, with MYD88- and CXCR4-driven dependence on BTK-NF-κB signaling. The pivotal iNNOVATE trial established ibrutinib–rituximab as superior to rituximab alone for PFS and depth of response, while the ASPEN study showed that zanubrutinib provides at least comparable efficacy with lower rates of atrial fibrillation and fewer off-target toxicities than ibrutinib [90,91,92].

BTK pathway targeting has also been explored in follicular lymphoma (FL) and diffuse large B-cell lymphoma (DLBCL), although the clinical impact has been more limited. In R/R FL, single-agent ibrutinib produced only modest activity in a phase II study of indolent NHL: the overall response rate (ORR) in the FL cohort was ~37% with a CR rate of ~12% and a median PFS of ~14 months, clearly inferior to results seen in marginal zone lymphoma or CLL [93]. Early phase combinations such as zanubrutinib plus obinutuzumab have yielded higher response rates in R/R FL (ORR ~68%, CR ~45%). Still, these data remain preliminary and have not yet been translated into guideline-defining roles for BTK inhibition in FL, where PI3K inhibitors, bispecific antibodies, and CAR-T products now dominate the relapsed space [94].

In DLBCL, BTK inhibition is biologically most relevant in ABC/non-GCB subtypes, but randomized data have been disappointing. The PHOENIX trial of ibrutinib plus R-CHOP versus R-CHOP alone in newly diagnosed non-GCB DLBCL did not improve event-free or overall survival in the intent-to-treat population; a signal of benefit was observed only in patients < 60 years, whereas older patients experienced greater toxicity and higher rates of treatment discontinuation [95]. Multiple phase II efforts are evaluating BTK inhibitors in combination with chemo-immunotherapy or immunomodulatory agents (e.g., zanubrutinib–rituximab–lenalidomide, ibrutinib + R-mini-CHOP in frail DLBCL), but these remain investigational [96,97,98]. An important niche exception is primary CNS lymphoma (PCNSL), where ibrutinib monotherapy has shown ORRs around 70% and CR ~50% in relapsed/refractory disease, with some patients achieving durable remissions, supporting BTK pathway dependence in this compartment and ongoing trials of BTKi-based combinations [99,100].

In routine clinical practice, the positioning of BTK inhibitors in indolent lymphoma follows disease-specific international guidelines. MCL first-line treatment in fit patients typically consists of chemoimmunotherapy, with autologous transplantation in selected cases, whereas BTK inhibitors are standard options in the relapsed or refractory setting. In MZL, anti-CD20–based immunochemotherapy or rituximab monotherapy represents a common first-line strategy, with covalent BTK inhibitors reserved for patients with disease progression or intolerance to initial therapy. In both entities, subsequent relapses after BTK inhibition are increasingly managed with alternative targeted agents and/or cellular therapies within clinical trials, underscoring that BTK inhibitors are embedded in broader, guideline-driven treatment algorithms rather than used in isolation.

Outside B-cell lymphomas, BTK is also expressed in mast cells and myeloid cells, and its inhibition attenuates FcεRI-dependent activation and downstream mediator release, providing a mechanistic rationale for exploring BTKi in mast-cell–driven disorders such as systemic mastocytosis and severe allergic phenotypes [101,102,103]. Early clinical experience with ibrutinib in mastocytosis and related conditions suggests biological activity on mediator-related symptoms, but responses are heterogeneous, and KIT-directed tyrosine-kinase inhibitors (e.g., midostaurin, avapritinib) remain the standard of care; BTKi use in this setting is therefore investigational and generally confined to clinical trials or highly selected, refractory cases [104,105,106,107]. Finally, in the allo-transplant setting, the dual BTK/ITK inhibition profile of ibrutinib has been leveraged to modulate both B-cell and T-cell signaling in chronic graft-versus-host disease (cGVHD). Ibrutinib is FDA-approved for steroid-refractory cGVHD after failure of at least one prior line of systemic therapy, especially after JAK inhibitor (ruxolitinib) failure, where it induces clinically meaningful, often durable responses in a sizeable proportion of patients and can restore corticosteroid sensitivity [108,109,110,111,112,113,114].

Finally, in immune thrombocytopenia (ITP), BTK inhibitors are being explored to interfere with Fcγ receptor– and BCR-mediated autoantibody production and platelet clearance. The oral reversible BTKi rilzabrutinib demonstrated promising early activity in phase 1/2 studies, with rapid, clinically relevant platelet responses in a subset of heavily pretreated patients. In the phase 3 LUNA3 trial (previously treated persistent/chronic ITP), rilzabrutinib achieved a durable platelet response (≥50 × 10^9^/L for ≥2/3 of ≥8 of the last 12 weeks without rescue) in 23% of patients versus 0% with placebo, with a median time to first response of 15 days [115].

## 6. Safety Profile, Toxicity Management, and Treatment Continuation

Class-specific toxicities of covalent BTKi include bleeding and bruising, infections, hypertension, and atrial fibrillation or flutter, with the relative frequency and severity of these events varying by agent and reflecting differences in kinase selectivity. Across significant phase II–III datasets, any-grade bleeding (including minor bruising and petechiae) is reported in roughly 30–40% of patients on ibrutinib, with major bleeding in about 2–5%, whereas second-generation agents such as acalabrutinib and zanubrutinib appear to be associated with somewhat lower rates of clinically significant hemorrhage [116,117,118,119]. In daily practice, preventive measures include a careful baseline assessment of concomitant antiplatelet or anticoagulant therapy, avoidance of triple antithrombotic regimens whenever possible, and temporary interruption of BTKi for 3–7 days before and after invasive procedures, with individualized decisions in patients at very high thrombotic risk.

Infections of any grade occur in more than half of patients over the course of therapy, and grade ≥ 3 infections in approximately 20–30%, with the highest incidence typically seen during the first 6–12 months of treatment, associated or not with grade 3/4 neutropenia [120,121,122]. Supportive care commonly includes vaccination against influenza, pneumococcus, and (where available) zoster before or early during treatment; prompt evaluation and empiric antibiotics for febrile episodes; and Pneumocystis jirovecii or antiviral prophylaxis in selected high-risk patients. In those with severe hypogammaglobulinemia and recurrent infections, immunoglobulin replacement may be considered. Diarrhea and arthralgia are also frequent but usually low-grade and manageable, whereas rash and transaminase elevations are less common and rarely dose-limiting [123]. Persistent grade ≥ 3 non-hematologic toxicity typically prompts dose reduction or switching to an alternative BTKi.

Cardiovascular toxicity is the most distinctive safety signal of the class and is particularly pronounced with first-generation ibrutinib. Across trials and real-world cohorts, atrial fibrillation has been reported in up to 15–20% of ibrutinib-treated patients at 3–5 years, with some series approaching 25% with longer follow-up, and clinically relevant hypertension in roughly 20–30%, including grade ≥ 3 hypertension in around 8–10%. In contrast, second-generation BTKi have consistently shown lower cardiovascular event rates while maintaining efficacy similar to or superior to that of first-generation BTKi. In ELEVATE-RR, acalabrutinib was noninferior to ibrutinib for PFS yet produced markedly fewer key cardiovascular events. Atrial fibrillation/flutter occurred in 9.4% of patients on acalabrutinib versus 16.0% on ibrutinib, and hypertension in 9.4% versus 23.2%, with fewer treatment discontinuations due to adverse events. In ALPINE, zanubrutinib not only improved efficacy endpoints compared with ibrutinib but also reduced atrial fibrillation/flutter to 7.1% versus 17.0%, with a lower overall incidence of cardiac events. Meta-analytic data and long-term extensions of pivotal studies support a consistent pattern in which second-generation BTKi reduce the relative risk of atrial fibrillation and major bleeding compared with ibrutinib. Overall, approximately 15–23% of patients on ibrutinib discontinue therapy for toxicity in long-term series, whereas discontinuation rates for intolerance appear lower with acalabrutinib and zanubrutinib, reflecting both improved tolerability and more favorable cardiovascular profiles [120,124,125].

Drug–drug interactions are another critical layer. In fact, proton-pump inhibitors can significantly reduce acalabrutinib absorption and should be avoided or appropriately spaced, and potent CYP3A inhibitors or inducers may necessitate BTKi dose modifications or alternative co-medications [126]. Finally, in the setting where BTKi are combined with venetoclax, TLS prophylaxis and stepwise dose ramp-up remain essential. When venetoclax is added, the TLS profile is driven mainly by venetoclax dose, tumor burden, and renal function rather than by BTKi exposure per se [127,128].

## 7. Conclusions: Future Directions and Evidence Gaps

Looking ahead, several cross-cutting themes are poised to redefine how BTK inhibition is deployed across hematologic diseases. In CLL/SLL, MCL, MZL, and WM, the central question is no longer whether BTK inhibitors are effective, but how to optimally integrate them with other targeted agents, antibodies, and cellular therapies. Fixed-duration and MRD-directed strategies with covalent BTKi plus BCL2 inhibitors or anti-CD20 antibodies are already well established in CLL and are beginning to be explored in MCL and WM; whether similar time-limited paradigms can safely replace lifelong BTK inhibition in these entities, without compromising long-term disease control or fostering more complex resistance, remains a key open question. Parallel efforts are needed to define rational sequencing between covalent and non-covalent BTKi, particularly in patients who have already exhausted BTK and BCL2 classes, and to understand how BTK pathway pressure interacts with subsequent therapies such as CAR T-cell products and bispecific antibodies.

In aggressive and indolent lymphomas beyond CLL/MCL/MZL/WM, the future role of BTK inhibition is less clearly defined and will depend on better biologic stratification. In DLBCL, where single-agent BTKi activity has been modest and confined to specific molecular subsets, BTK pathway blockade is more likely to serve as a component of rational combinations (for example, with chemoimmunotherapy, PI3K/AKT pathway inhibitors, or immune effectors) rather than as a standalone backbone. Systematic integration of genomic and transcriptomic profiling will be essential to identify those niches in which BTK dependence is sufficiently strong to justify ongoing clinical development. In parallel, non-malignant indications such as GVHD and ITP illustrate how BTK inhibition can modulate Fc receptor signaling, autoantibody production, and innate immune activation; expanding this paradigm to other autoantibody-mediated or alloimmune disorders will require disease-specific mechanistic work and carefully designed phase 2–3 programs.

Across all these settings, several overarching gaps persist. Standardized, disease-agnostic approaches to MRD assessment and resistance monitoring (BTK/PLCG2 and non-canonical kinase-domain lesions, as well as parallel pathway activation) are still lacking. Still, they are critical to moving from empiric line-by-line usage toward biology-guided sequencing. Long-term cardiovascular and infectious safety of chronic BTK inhibition remains incompletely characterized and demands dedicated cardio-oncology and infection-risk stratification tools rather than simple extrapolation from early CLL cohorts. Finally, comparative effectiveness data between covalent and non-covalent BTKi and between BTK-based and non-BTK-based strategies (e.g., BCL2-, CAR-T- or bispecific-anchored pathways) are still limited outside CLL.

Finally, a broader pipeline of BTK-directed therapies is in active development. Additional covalent inhibitors such as tirabrutinib, orelabrutinib, and rilzabrutinib have been designed to enhance BTK selectivity and/or modulate reversibility of C481 binding, with early signals of activity across B-cell malignancies and autoimmune indications, including immune thrombocytopenia. Nemtabrutinib (MK-1026) and vecabrutinib (non-covalent BTKi) seek to broaden coverage of BTK-domain variants and to minimize off-target TEC family inhibition.

A conceptually distinct strategy is the targeted proteasomal degradation of BTK. Heterobifunctional BTK degraders such as NX-2127, NX-5948, and BGB-16673 couple high-affinity BTK engagement to E3 ligase recruitment, promoting ubiquitination and proteasomal elimination of both wild-type and mutant BTK. Early phase data indicate clinically meaningful BTK degradation and responses in heavily pretreated, covalent BTKi-exposed cohorts, but longer follow-up is needed to clarify durability, off-target immunomodulation, and safety, particularly concerning infection risk and cytopenias.

## Figures and Tables

**Table 1:** Evidence from first-line clinical trials.

TRIAL	Arms	Key Results
RESONATE-2	Ibrutinib 420 mg QD (continuous) vs. chlorambucil (≤12 cycles)	Median PFS 8.9 years vs. 1.3 years; median OS not reached with ibrutinib; benefit preserved across genomic risk; many patients still on ibrutinib at 8–10 years
Alliance A041202	BR vs. ibrutinib vs. ibrutinib–rituximab	Both ibrutinib-containing arms improved PFS vs. BR; no additional PFS benefit from adding rituximab; long-term benefit primarily in unmutated IGHV
E1912	Ibrutinib–rituximab vs. FCR	3-year PFS 89.4% vs. 72.9% (HR ~0.35); 3-year OS 98.8% vs. 91.5% (HR ~0.17); benefit regardless of IGHV status
ELEVATE-TN	Acalabrutinib–obinutuzumab vs. acalabrutinib vs. chlorambucil–obinutuzumab	At 6 years: PFS 78.0% (A + O) vs. 61.5% (A) vs. 17.2% (Clb + O); median PFS not reached in acalabrutinib arms; benefit maintained in high-risk subsets (del17p/TP53, unmutated IGHV, complex karyotype)
SEQUOIA	Zanubrutinib vs. BR	2-year PFS 85.5% vs. 69.5%; ~5-year follow-up: sustained PFS separation; 60-month OS ~86% in both arms; AF ~7% with zanubrutinib; separate nonrandomized del(17p) cohort showed durable control
CAPTIVATE	3 cycles ibrutinib lead-in → 12 cycles ibrutinib + venetoclax	CR ~55–56%; PB uMRD ~77%; 24-mo PFS ~95%, 24-mo OS ~98%; high-risk subgroups: 36-mo PFS ~88–92%, OS > 95%
CLL17	Continuous ibrutinib vs. fixed-duration venetoclax–obinutuzumab vs. fixed-duration venetoclax–ibrutinib	3-year PFS 81.1% (Ven + O) vs. 79.4% (Ven + Ibr) vs. 81.0% (Ibr): both fixed-duration arms noninferior; post-treatment PB uMRD 73.3% vs. 47.2% vs. 0%; 3-year OS 91.5–96.0%
AMPLIFY	Acalabrutinib–venetoclax vs. Acalabrutinib–venetoclax–obinutuzumab vs. CIT (FCR/BR)	At ~41 months: 36-mo PFS 76.5% (A + V) vs. 83.1% (AVO) vs. 66.5% (CIT); median PFS not reached in experimental arms vs. 47.6 mo with CIT; HR 0.65 (A + V vs. CIT) and 0.42 (AVO vs. CIT); OS > 85% across arms
MRD-guided phase 2 (NCT03580928)	Sequential Acalabrutinib → + Obinutuzumab → +Venetoclax; MRD-guided stopping (15 or 24 cycles if uMRD)	*n* = 72 (45 TP53-aberrant): CR with BM-uMRD at cycle 16 = 42% (TP53-aberrant and overall); BM-uMRD 71% (TP53-aberrant)/78% overall; median follow-up 55.2 mo: 4-year PFS/OS 70%/96% (TP53-aberrant) and 88%/100% (non–TP53-aberrant)
FLAIR	CIT vs. continuous ibrutinib vs. MRD-directed ibrutinib–venetoclax	At 5 years: ~94% progression-free with Ibr + Ven vs. ~79% with ibrutinib alone vs. ~58% with CIT; ~two-thirds achieved BM uMRD after 2 years in the combination arm

QD: once a day; PFS: progression-free survival; OS: overall survival: BR: bendamustine-rituximab; AF: atrial fibrillation; uMRD: undetectable minimal residual disease; CIT: chemoimmunotherapy; BM: bone marrow; mo: months.

**Table 2:** Evidence from further-line clinical trials.

TRIAL	Arms	Key Results
ELEVATE-RR	Acalabrutinib 100 mg BID (continuous) vs. ibrutinib 420 mg QD (continuous) in del(11q) and/or del(17p)	Median PFS 38.4 vs. 38.4 months (noninferiority; HR ~1.00). AF/flutter 9.4% vs. 16.0%; HTN 9.4% vs. 23.2%; bleeding and discontinuations due to AEs lower with acalabrutinib
ASCEND	Acalabrutinib 100 mg BID (continuous) vs. investigator’s choice: idelalisib–rituximab or BR	Median PFS not reached vs. 16.8 months (HR 0.31). 42-month PFS 62% vs. 19% (acalabrutinib vs. comparator)
RESONATE	Ibrutinib 420 mg QD (continuous) vs. ofatumumab (infusional)	Median PFS 44.1 vs. 8.1 months; OS benefit reported; efficacy retained in high-risk subgroups
ALPINE	Zanubrutinib 160 mg BID (continuous) vs. ibrutinib 420 mg QD (continuous)	Superior PFS: HR 0.68; 3-year PFS 64.9% vs. 54.8%. ORR 85.6% vs. 75.4%. AF/flutter 7.1% vs. 17.0%; fewer overall cardiac events with zanubrutinib
BRUIN CLL-321	Pirtobrutinib vs. investigator’s choice: idelalisib–rituximab or bendamustine–rituximab post-covalent BTKi	Pirtobrutinib improved PFS and time-to-next-treatment vs. comparator in a heavily pretreated population enriched for C481-mediated resistance; supports use as a non-covalent BTKi “bridge” after covalent BTKi (and commonly after BCL2i exposure)

QD: once a day; HR: hazard ratio; HTN: hypertension; AEs: adverse effects; PFS: progression-free survival; OS: overall survival: BR: bendamustine-rituximab; AF: atrial fibrillation; ORR: overall response ratio.

## Data Availability

No new data were created or analyzed in this study.
